# The associations of dietary folate and serum folate with lipid profiles: findings from the national health and nutrition examination survey 2011–2016

**DOI:** 10.1186/s12944-023-01793-4

**Published:** 2023-03-02

**Authors:** Yunfu Feng, Xiaohua Chen, Ying Pan, Yanting Yang

**Affiliations:** 1 Department of General Practice, First Peopleple’s Hospital of Kunshan, Kunshan, 215300 China; 2 Department of Gastroenterology, Third Peopleple’s Hospital of Kunshan, Kunshan, 215300 China

**Keywords:** Folate, Lipid profiles, NHANES, A cross-sectional study

## Abstract

**Background:**

Folate is considered to be related to lipid metabolism. With the increasing numbers of folic acid fortification nations, the associations of dietary folate and serum folate with lipid profiles deserve more attention and are worth further study.

**Methods:**

US adults aged ≥ 20 years from the National Health and Nutrition Examination Survey (NHANES) were evaluated. Participants taking folic acid supplements were excluded. The multivariate linear regression model and smooth curve fitting were applied to assess the associations. The segmented regression model was employed to examine the threshold effect of nonlinear relationships.

**Results:**

Our cross-sectional study included 3706 participants in total. There was a negative relationship between serum folate (log transformed) and triglycerides (β = –0.223, 95% CI: –0.337, –0.110) and low-density lipoprotein cholesterol (LDL-C) (β = –0.152, 95% CI: –0.296, –0.007) and a positive relationship between serum folate (log transformed) and high-density lipoprotein cholesterol (HDL-C) (β = 0.090, 95% CI: 0.033,0.146). There was a negative association between dietary folate (log transformed) and total cholesterol (TC) (β = –0.299, 95% CI: –0.465, –0.134) and LDL-C (β = –0.266, 95% CI: –0.409, –0.123). A nonlinear relationship was found between dietary folate (log transformed) and HDL-C. Threshold effect analysis showed that the inflection point was 377.57 ug. Within the inflection point, the β-coefficient of HDL-C was 0.105 (95% CI: 0.018, 0.192); beyond the inflection point, there was no relationship (β = –0.067, 95% CI: –0.162, 0.028).

**Conclusions:**

Optimal dietary folate and high serum folate were associated with favorable lipid profiles. Dietary folate, in the recommended 300–400 ug/d, had a beneficial effect on improving lipid profiles.

**Supplementary Information:**

The online version contains supplementary material available at 10.1186/s12944-023-01793-4.

## Background

Folate (vitamin B9) is a fundamental B vitamin needed to maintain one-carbon transfer reactions. One-carbon metabolism requires all B vitamins to serve as cofactors; however, folate is a unique catalytic substrate involved in one-carbon transfer reactions such as DNA methylation and biosynthesis of nucleic acids and amino acids [1, 2]. Folate deficiency is related to various diseases, such as anemia or neural tube defects (NTDs) [3], and leads to hyperhomocysteinemia, a high-risk factor for ischemic heart disease, stroke, and depression [4–6]. Disorders of lipid metabolism are a frequent health problem. Plasma lipid profiles contain triglycerides (TG), total cholesterol (TC), low-density lipoprotein cholesterol (LDL-C), and high-density lipoprotein cholesterol (HDL-C). Deoxyribonucleic acid (DNA) methylation influences lipoprotein profiles and lipid metabolism-related diseases [7].

Some studies have emphasized the relationship between folic acid and lipid profiles, especially focusing on folic acid supplementation (FAS). A systematic review by Reza et al. [8] highlighted that FAS did not influence plasma lipoprotein levels among metabolic-related disease patients. Asbaghi et al. [9] indicated that FAS diminished serum triglycerides and TC concentrations but had no influence on LDL-C or HDL-C. To date, only a few reports have focused on serum folate and plasma lipids. A retrospective study in Germany found that high serum folate was related to low LDL-C and high HDL-C [10]. Another report from a Danish population database demonstrated that serum folate was positively related to HDL-C but negatively associated with LDL-C [11]. In the Swedish Apolipoprotein MOrtality RISk (AMORIS) study, TG, TC, and folate levels were not correlated [12]. As these studies included a population that was predominantly Caucasian, the results may not apply to all populations.

A large sample database from the US, a multiracial and multicultural nation, provides a representative wider population. After two decades of folic acid fortification, folate levels became stable in the US population over the period 2011 to 2016 [13]. Globally, 91 nations agreed on the folic acid fortification of industrially milled cereal grains (i.e., cereal, bread, rice, and pasta) in 2021 (https://www.ffinetwork.org/annual-reports), considering that a significant correlation was demonstrated between dietary and serum folate [14, 15]. Therefore, our cross-sectional study assessed the associations of serum folate and dietary folate with lipid profiles among US adults aged ≥ 20 years from the National Health and Nutrition Examination Survey (NHANES).

## Methods

### Study population

The NHANES is a national survey coordinated by the Centers for Disease Control and Prevention (CDC) that monitors the health and nutritional status of the entire US population. The study was approved by the ethics review board of the National Center for Health Statistics (NCHS), and all participants signed an informed consent [16].

Our study population contained three cycles of the NHANES between 2011 and 2016. After excluding missing lipid profiles (*N* = 14,914), missing serum folate (*N* = 6779), missing dietary folate (*N* = 455), age < 20 years (N = 1529), intake of medicines that affect serum folate or lipid profiles such as methotrexate, fenofibrate, and statins (*N* = 1432), pregnant women (*N* = 244), and folic acid supplements participants (*N* = 848), this study included 3706 participants in total.

### Variables

The exposure variables were serum folate and dietary folate. Serum folate measured by isotope-dilution high-performance liquid chromatography coupled to tandem mass spectrometry in the NHANES laboratory was calculated by adding 5-methyl-tetrahydrofolate, pteroylglutamic acid, 5-formyl-tetrahydrofolate, tetrahydrofolate, and 5,10-methenyl-tetrahydrofolate. An overnight fast was advised before specimen collection. Information on dietary folate intake from the participants of NHANES was obtained through a 24-h dietary interview. An estimation of food and beverage consumption during the 24 h before the interview was conducted to assess dietary folate intake. Dietary folate included folic acid in natural foods and in folic acid-fortified foods. Dietary folate equivalents (DFE) were used to assess dietary folate intake because of the differential bioavailability of folic acid in natural foods and synthetic folic acid. The outcome variable was plasma lipid profiles containing TG, TC, HDL-C, and LDL-C.

### Covariates

The continuous covariates were age, body mass index (BMI), energy intake, total fat intake and dietary fiber intake. The categorical covariates were sex, ethnicity, education level, smoking habits, alcohol consumption, diabetes and hypertension [17]. Food energy, nutrients, and food components from food or beverages as calculated using the Food and Nutrient Database of the U.S. Department of Agriculture for Dietary Studies. The 24-h dietary interview yielded data on energy intake, total fat intake, and dietary fiber intake. Ethnicity was classified as non-Hispanic white, non-Hispanic black, Mexican American, and other race. Less than 12th grade, high school graduate or college degree, and college graduate or above were the education levels. Based on questionnaire data, smoking habits were divided into current, former and never; alcohol consumption was divided into drinker or nondrinker. Hypertension and diabetes were self-reported.

### Statistical analyses

The continuity variable was reported as the mean ± standard deviation (SD), and the categorical variable was reported as percentages. Because serum folate and dietary folate were not normally distributed, we used log transformation for statistical analysis. The multivariate linear regression model was used to assess the associations between serum and dietary folate and lipid profiles. Age, sex, race, education level, BMI, energy intake, total fat intake, dietary fiber intake, smoking habits, alcohol consumption, diabetes and hypertension were considered potential factors. Smooth curve fitting and a generalized additive model were conducted to resolve the nonlinear relationship. The segmented regression model was used to examine the threshold effect of the nonlinear relationship between folate and lipid profiles. A two-sided *P* < 0.05 indicated a statistically significant difference. All statistical data were analyzed using R (http://www.R-project.org, The R Foundation, Boston, MA, USA) and EmpowerStats software (http://www.empowerstats.com, X&Y Solutions, Inc., Boston, MA, USA).

## Results

A summary of the basic characteristics of the study population is illustrated in Table 1. This study involved 3706 participants, comprising 1799 males and 1907 females. The mean serum folate was 34.87 ± 15.60 nmol/L, and the mean dietary folate (DFE) was 477.58 ± 265.04 ug, of which food folate was 216.00 ug and synthetic folic acid was 153.87 ug. The positive relationship between dietary folate (log transformed) and serum folate (log transformed) (β = 0.206, 95% CI: 0.173,0.239) is shown in Supplementary Fig. 1. Within the inflection point (Lg dietary folate = 2.193, dietary folate = 155.96 ug), the β-coefficient of the segmented model was –0.084 (95% CI: –0.238, 0.071). Beyond the inflection point, the β-coefficient was 0.241 (95% CI: 0.203, 0.278). The threshold effect of dietary folate on serum folate was significant, log-likelihood ratio < 0.001. (Supplementary Table 1).

**Table 1:** Characteristics of the study population

*General characteristics*	
Age (years)	44.55 ± 16.33
Male (%)	48.54
Race/Ethnicity (%)	
Non-Hispanic White	35.59
Non-Hispanic Black	22.07
Mexican American	15.76
Other Race	26.58
Education level (%)	
Less than 12^th^ grade	23.63
High school graduate or College degree	52.52
College graduate or above	23.85
BMI	29.13 ± 7.19
Energy intake (kcal)	2118.42 ± 969.05
Total fat intake (g)	80.71 ± 45.19
Dietary fiber intake (g)	16.45 ± 10.34
Smoking habits (%)	
Current	21.96
Former	19.37
Never	58.66
Drinker (%)	53.32
Diabetes (%)	7.34
Hypertension (%)	27.70
*Exposures*	
Serum folate (nmol/L)	34.87 ± 15.60
Dietary folate, DFE (ug)	477.58 ± 265.04
*Outcomes*	
Triglycerides (mmol/L)	1.26 ± 0.68
Total cholesterol (mmol/L)	4.99 ± 0.99
HDL cholesterol (mmol/L)	1.38 ± 0.37
LDL cholesterol (mmol/L)	2.99 ± 0.85

*LDL* Low-density lipoprotein, *HDL* High-density lipoprotein, *DFE* Dietary folate equivalents

### Serum folate and lipid profiles

The results of the multivariate linear regression are shown in Table 2. There was a negative relationship between serum folate (log transformed) and triglycerides (β = –0.223, 95% CI: –0.337, –0.110) and LDL-C (β = –0.152, 95% CI: –0.296, –0.007) and a positive relationship between serum folate (log transformed) and HDL-C (β = 0.090, 95% CI: 0.033,0.146). Moreover, insignificant relationships existed between serum folate (log transformed) and TC.

**Table 2:** Association between serum folate (log transformed) and lipid profiles among US adults aged ≥ 20 years

Outcomes	Crude β (95% CI)	Adjust β I (95% CI)	Adjust β II (95% CI)
Serum folate (log transformed)			
Triglycerides (mmol/L)	–0.192 (–0.301, –0.083)	–0.251 (–0.358, –0.145)	–0.223 (–0.337, –0.110)
Total cholesterol (mmol/L)	0.106 (–0.051, 0.263)	–0.163 (–0.319, –0.007)	–0.143 (–0.309,0.023)
HDL cholesterol (mmol/L)	0.212 (0.154, 0.270)	0.090 (0.036, 0.144)	0.090 (0.033,0.146)
LDL cholesterol (mmol/L)	–0.062 (–0.197, 0.072)	–0.154 (–0.290, –0.019)	–0.152 (–0.296, –0.007)

Adjust I for age, sex, race, and BMI

Adjust II for age, sex, race, education level, BMI, energy intake, total fat intake, dietary fiber intake, smoking habits, alcohol consumption, diabetes, and hypertension

### Dietary folate (DFE) and lipid profiles

**Fig. 1:**
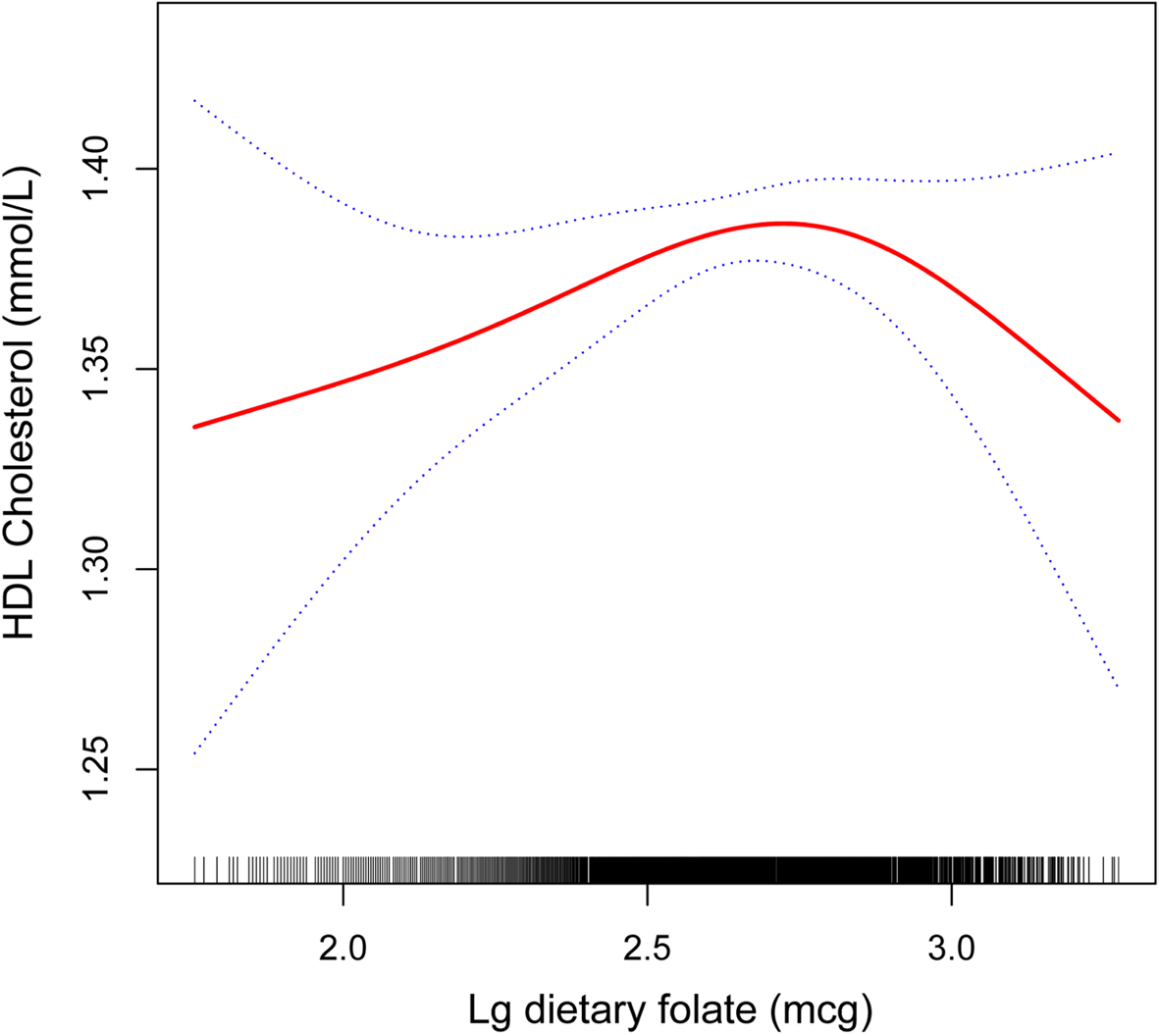
Association between dietary folate (log transformed) and HDL-C among US adults aged ≥ 20 years. The solid red line represents the relation between the variables, the blue dotted lines indicate a 95% confidence interval, and the black bars show frequency. Adjusted for age, sex, race, education level, BMI, energy intake, total fat intake, dietary fiber intake, smoking habits, alcohol consumption, diabetes, and hypertension

The results of the multivariate linear regression are shown in Table 3. There was a negative association between dietary folate (log transformed) and TC and LDL-C. There was no association between dietary folate (log transformed) and triglycerides. From smooth curve fitting analysis (Fig. 1), a nonlinear relationship was found between dietary folate (log transformed) and HDL-C. The threshold effect of dietary folate on HDL-C was observed in Table 4. Within the inflection point (Lg dietary folate = 2.577, dietary folate = 377.57 ug), a positive relationship was observed between dietary folate and HDL-C (β = 0.105, 95% CI: 0.018, 0.192). Beyond the inflection point, there was no relationship (β = –0.067, 95% CI: –0.162, 0.028).

**Table 3:** Association between dietary folate (log transformed) and lipid profiles among US adults aged ≥ 20 years

Outcomes	Crude β (95% CI)	Adjust β I (95% CI)	Adjust β II (95% CI)
Dietary folate (log transformed), DFE			
Triglycerides (mmol/L)	–0.042 (–0.127, 0.043)	–0.090 (–0.172, –0.008)	–0.108 (–0.221, 0.005)
Total cholesterol (mmol/L)	–0.273 (–0.395, –0.151)	–0.216 (–0.336, –0.096)	–0.299 (–0.465, –0.134)
HDL cholesterol (mmol/L)	–0.030 (–0.076, 0.016)	0.047 (0.005, 0.089)	0.025 (–0.032, 0.082)
LDL cholesterol (mmol/L)	–0.219 (–0.323, –0.114)	–0.211 (–0.315, –0.107)	–0.266(–0.409, –0.123)

Adjust I for age, sex, race, and BMI

Adjust II for age, sex, race, education level, BMI, energy intake, total fat intake, dietary fiber intake, smoking habits, alcohol consumption, diabetes, and hypertension

**Table 4:** Threshold effect analysis of dietary folate (log transformed) on HDL-C using the segmented regression model

HDL cholesterol	Adjust β (95% CI)
The standard linear model	0.025 (–0.032, 0.082)
The segmented model	
Inflection point (log transformed)	2.577
Dietary folate (log transformed) < 2.577	0.105 (0.018, 0.192)
Dietary folate (log transformed) > 2.577	–0.067 (–0.162, 0.028)
Log likelihood ratio	0.017

Adjusted for age, sex, race, education level, BMI, energy intake, total fat intake, dietary fiber intake, smoking habits, alcohol consumption, diabetes, and hypertension

## Discussion

In our study, the database of NHANES 2011–2016 was applied to examine the associations of dietary folate and serum folate with lipid profiles among US adults aged ≥ 20 years. There was a negative relationship between serum folate and triglycerides and LDL-C and a positive relationship between serum folate and HDL-C. Then, there was a negative association between dietary folate and TC and LDL-C. A nonlinear relationship was observed between dietary folate and HDL-C with inflection points. Altogether, optimal dietary folate and high serum folate were associated with favorable lipid profiles.

With an increasing number of nations using folic acid fortification, the relationships of serum folate and dietary folate with lipid profiles are worth evaluating, especially in the US, which has implemented folic acid fortification since 1998. Our study analyzed the associations between serum and dietary folate and lipid profiles. The mean serum folate concentration was 34.87 nmol/L, approximately twofold that in European studies [10, 11]. In a Danish population-based study including 12,532 adults, serum folate was positively correlated with HDL-C (β = 0.03, 95% CI: 0.02, 0.03) and inversely correlated with LDL-C (β = –0.03, 95% CI: –0.05, –0.01). The weighted genetic risk scores of folate were linked with increased HDL-C (β = 0.081, 95% CI: 0.015, 0.148) but not TG, TC, and LDL-C [11]. In an analysis of the hypertensive population in China, the low serum folate group exhibited high odds of hypertriglyceridemia (OR = 2.02, 95% CI: 1.25–3.25) and low levels of HDL-C (OR = 1.88, 95% CI: 1.07–3.28) compared with the normal serum folate group. Likewise, a weak positive relationship was found between folate and HDL-C (adjusted β = 0.14, *P* = 0.002) but not TG, TC, and LDL-C [18]. In our study, we discovered associations between serum folate and TG, HDL-C, and LDL-C but not TC. This correlation required observation over time to determine the causality.

Dietary folate and lipid profiles were also examined in our study. The mean dietary folate (DFE) was 477.58 ug, more than the Recommended Dietary Allowance (RDAs) of the US and World Health Organization (WHO) for folate, 400 ug/d (https://www.ncbi.nlm.nih.gov/books/NBK114318/, accessed on 31 July 2022). The European Food Safety Authority (EFSA) set the population reference folate intake at 330 ug/d and the average requirement at 250 ug/d [19]. The prevalence of folate deficiency has typically been higher among nations without folic acid fortification. Dietary folate in most European countries was much lower than the recommendation of 400 ug/d and even less than 300 ug/d [20]. In a Japanese population study, high dietary vitamin intake, including folic acid, was related to low mean adenosine triphosphate- (ATP)-binding cassette transporter A1 (ABCA1) DNA methylation levels. Meanwhile, an inverse correlation was found between mean ABCA1 DNA methylation and HDL cholesterol [21]. Mohammadian et al. [22] found that using folic acid can be beneficial in treating dyslipidemia caused by cholestasis. High folate intake might be beneficial for lipid profiles. However, a concern was raised concerning high dietary folate intake related to the potential for increased risk of cancer, including colorectal and prostate cancers [23, 24]. In addition, recent animal studies revealed that high FAS intake might cause epigenetic alterations in offspring, increasing the risk of diabetes and changing food intake behavior [25]. Kelly et al. [26] suggested that excess FAS intake compared to adequate FAS may exacerbate weight gain and fat accumulation in rats on high-fat diets. In conclusion, excessive or too little folic acid may result in adverse effects, and the results in this study supported the optimal dietary folate related to favorable lipid profiles.

The relationships between folic acid and lipid metabolism have been observed. Folate deficiency impairs methylation capacity and reduces de novo phosphatidylcholine synthesis, leading to nonalcoholic fatty liver disease and obesity [1]. Christensen et al. [27] found that a deficiency of folate in mice induced hepatic steatosis. Later, they observed how high FAS caused pseudomethylenetetrahydrofolate reductase deficiency, altered lipid metabolism, and hepatic damage in mice [28]. When studying the mechanisms between folate and lipids, epigenetic modifications such as DNA methylation may play an important role. Various factors can influence epigenetic modification, including alleles, type of cell, and phase of development [7, 21]. Environmental factors, such as diet and pollution exposure, can also affect the methylation of lipid-related genes. As observed in the study, FAS modified the phenotype and epigenotype in young rats [29]. The possible mechanism by which folate influences plasma lipids is related to various factors, genetic, epigenetic and environmental, and even then, the relationship between folate and lipids still cannot be fully explained. However, the role of folate requires further research as a critical intermediate link, enhancing human health.

### Study strengths and limitations

Notably, the major strengths of this study should be considered. First, based on the NHANES database, a large population (3706 subjects) was included, with nationally representative samples among US adults. Considering the racial mixture in the US, our results can represent a general population receiving folic acid fortification. All data obtained from the NHANES database were obtained by standard procedures and trained staff, which provides accuracy and effectiveness of the study. Second, strict exclusion criteria were used in our study; medications such as methotrexate, fenofibrate, and statins that affect serum folate or lipid profiles were excluded. Third, we analyzed smooth curve fittings and threshold effects and found the turning point of folate on HDL-C. Nevertheless, limitations existed. First, given the cross-sectional nature of the study, our study did not establish a cause and effect. Second, our study did not involve homocysteine, which is closely related to folate but may not be linked to lipids. The results showed that serum homocysteine was not independently associated with lipids in the Very Large Database of Lipids-21 study [30] and a Chinese hypertensive population study [18]. Third, our study did not enroll FAS as a dependent variable or covariate. The missing data of FAS in NHANES occupied a larger proportion. However, this study covered folic acid fortification, which is also a form of FAS, and encompassed a larger population.

## Conclusions

Optimal dietary folate and high serum folate were associated with favorable lipid profiles. Inflection points of the nonlinear relationship were found between dietary folate and HDL-C. The advantage was observed in the lipid profiles in the current folic acid fortification strategy implemented in the US. Dietary folate, in the recommended 300–400 ug/d, had a beneficial effect on improving lipid profiles.

## Supplementary Information

**Additional file 1:** **Supplementary Figure 1.** Association between dietary folate (log transformed) and serum folate (log transformed). The solid red line represents the relation between the variables, the blue punctate lines indicate a 95% confidence interval, and the black bars show frequency. Adjust for: age, gender, race, education level, BMI, energy intake, total fat intake, dietary fiber intake, smoking habits, alcohol consumption, diabetes, hypertension. **Supplementary Table 1.** Threshold effect analysis of dietary folate on serum folate, using the segmented regression model.

## Data Availability

The database used for this study can be found in online repositories. For more information, visit https://www.cdc.gov/nchs/nhanes/index.htm.

## Abbreviations

NHANES: The national health and nutrition examination survey
TG: Triglycerides
TC: Total cholesterol
HDL-C: High-density lipoprotein cholesterol
LDL-C: Low-density lipoprotein cholesterol
FAS: Folic acid supplementation
CI: Confidence interval
BMI: Body mass index
DFE: Dietary folate equivalents
