# Supplementary material for: The associations of dietary folate and serum folate with lipid profiles: findings from the national health and nutrition examination survey 2011–2016
Source: Lipids Health Dis. 2023 Mar 2;22:30. doi: 10.1186/s12944-023-01793-4 (PMC9979480; doi:10.1186/s12944-023-01793-4)
Supplement: Supplementary file 1 — Additional file 1: Supplementary Figure 1. Association between dietary folate (log transformed) and serum folate (log transformed). The solid red line represents the relation between the variables, the blue punctate lines indicate a 95% confidence interval, and the black bars show frequency. Adjust for: age, gender, race, education level, BMI, energy intake, total fat intake, dietary fiber intake, smoking habits, alcohol consumption, diabetes, hypertension. Supplementary Table 1. Threshold effect analysis of dietary folate on serum folate, using the segmented regression model. [file 12944_2023_1793_MOESM1_ESM.docx]

**Supplementary material**


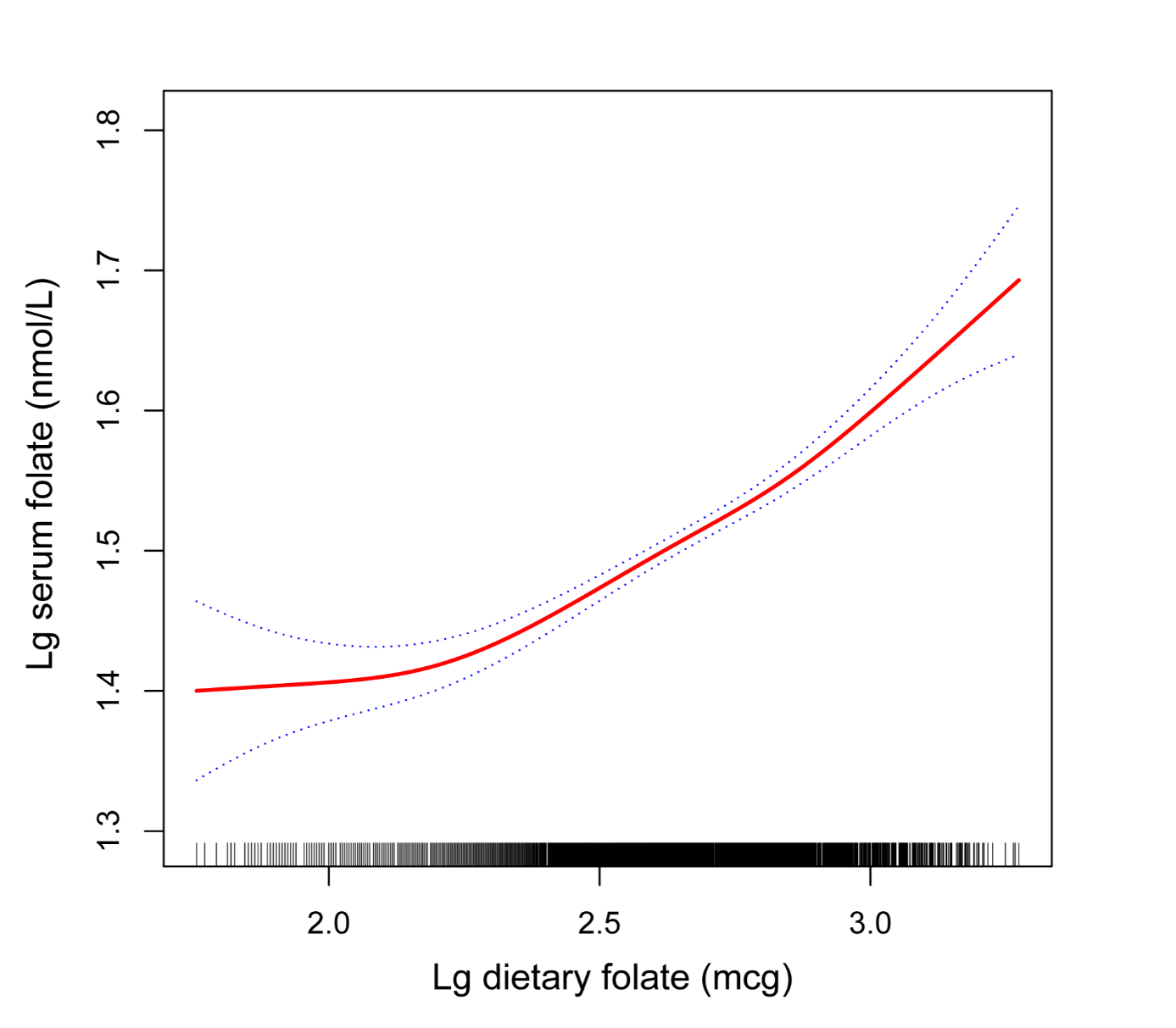


**Supplementary Figure 1**: Association between dietary folate (log transformed) and serum folate (log transformed). The solid red line represents the relation between the variables, the blue punctate lines indicate a 95% confidence interval, and the black bars show frequency. Adjust for: age, gender, race, education level, BMI, energy intake, total fat intake, dietary fiber intake, smoking habits, alcohol consumption, diabetes, hypertension.

**Supplementary Table 1. Threshold effect analysis of dietary folate on serum folate, using the segmented regression model.**

| Serum folate (log transformed) | Adjust β (95% CI) |
| --- | --- |
| The standard linear model | 0.206 (0.173, 0.239) |
| The segmented model |  |
| Inflection point (log transformed) | 2.193 |
| Dietary folate (log transformed) < 2.193 | –0.084 (–0.238, 0.071) |
| Dietary folate (log transformed) > 2.193 | 0.241 (0.203, 0.278) |
| Log likelihood ratio | <0.001 |

Adjust for: age, gender, race, education level, BMI, energy intake, total fat intake, dietary fiber intake, smoking habits, alcohol consumption, diabetes, hypertension.
